# Rapid and sensitive detection of tetracycline residue in food samples using Cr(III)-MOF fluorescent sensor

**DOI:** 10.1016/j.fochx.2023.100883

**Published:** 2023-09-16

**Authors:** Arezou Khezerlou, Milad Tavassoli, Mahmood Alizadeh Sani, Zahra Ghasempour, Ali Ehsani, Balal Khalilzadeh

**Affiliations:** a Student Research Committee, Department of Food Science and Technology, Faculty of Nutrition and Food Science, Tabriz University of Medical Sciences, Tabriz, Iran; b Biotechnology Research Center, Tabriz University of Medical Sciences, Tabriz, Iran; c Division of Food Safety and Hygiene, Department of Environmental Health Engineering, School of Public Health, Tehran University of Medical Sciences, Tehran, Iran; d Department of Food Science and Technology, Faculty of Nutrition and Food Sciences, Tabriz University of Medical Sciences, Tabriz, Iran; e Stem Cell Research Center (SCRC), Tabriz University of Medical Sciences, Tabriz 51666-14711, Iran

**Keywords:** Fluorescence sensor, Food analysis, Residual antibiotics, Metal-organic frameworks, Optical probes

## Abstract

As tetracycline antibiotics were used in the poultry sector, their residue in edible animal products may adversely affect food safety and human health. The development of selective and sensitive tetracycline sensors has garnered a lot of interest due to the complexity of food samples. Therefore, a fluorescent sensing probe based on chromium(III)-metal–organic framework was developed for the rapid detection of tetracycline. After the addition of tetracycline, blue emission at λem 410 nm was effectively quenched by the interaction between TC and Cr(III)-metal–organic framework material. Under optimized conditions (sensor concentration: 30 mg/L and pH: 10.0), the sensing probe showed a fast response time (1 min), and low detection limit (0.78 ng/mL) with a linear range (5–45 ng/mL). Interestingly, the Cr(III)-metal–organic framework was successfully applied to quantity tetracycline residue in chicken meat and egg samples with recoveries of 95.17–06.93%. To deduce, our work can provide a new strategy for the direct detection of tetracycline in food samples.

## Highlights

• A Cr(III)-MOF fluorescent sensor was developed to detect of tetracycline antibiotic.
• The FL-based sensor showed high sensitivity and selectivity toward tetracycline.
• The FL-based sensor detected tetracycline with a low detection limit = 0.78 ng/mL.
• The FL-based sensor successfully applied to analysis of tetracycline in food samples.

## 1 Introduction

Tetracycline (TC) is a bacteriostatic drug that is widely administered for the prevention or treatment the various infectious diseases in animals, and humans (Tariq et al., 2018). In addition, TC as a feed additive is extensively used in the livestock and aquaculture sectors for growth promotion (Granados-Chinchilla & Rodriguez, 2017). Since this antibiotic is hard to digest by animals and humans, TC is excreted in relatively large quantities into soil, water, and sewage, and its improper administration and indiscriminate use may lead to the accumulation of TC in animal-based food products like meat muscles, egg, honey, and milk (Pattanayak et al., 2022). The spreading of antibiotic-resistant strains and other side effects of TC residue in food products have become an intense concern for public health. The tolerable daily intake of TC set by the Food and Drug Administration is 0.25 mg/kg body weight/day (Bahmani et al., 2020). According to the European Union (EU), TC’s maximum residue limit is 100 and 200 µg/kg for chicken meat and egg, respectively (Baghani et al., 2019, Yang et al., 2021). On this account, to monitor the trace levels of antibiotic residues in environmental samples and food matrices, a very sensitive and efficient analytical approach is required.

The numerous analytical methods, including chromatographic (High-pressure liquid chromatography (HPLC) (Galyautdinova et al., 2020), and liquid chromatography (LC)) with/without mass spectrometric methods (Feng et al., 2016), capillary electrophoresis (Colombo & Papetti, 2019), spectrofluorometric (Ehtesabi et al., 2019), electrochemical (Mansouri et al., 2020, Raykova et al., 2021) and antibody-based methods (Baghani et al., 2019, Nasrollahpour et al., 2021), have been employed for the quantitative and qualitative detection of TC. Although all methods have good precision and accuracy, their applications are limited in routine analysis due to costly equipment, time-consuming, and often need complicated testing stages and well-skilled technicians (Chenaghlou et al., 2021). Accordingly, developing suitable strategies with low-cost, high performance, sensitivity, and selectivity, reliable, and simple implementation towards TC detection is desirable.

In recent decades, fluorescence (FL)-based sensors have shown huge attention as detection methods in light of their easy-to-operate, low-cost, time saving and high detection sensitivity (Gan et al., 2021, Gan et al., 2021, Nasrollahpour et al., 2022). The self-assembled structures (containing metal clusters/ions nodes and organic linkers) of metal–organic frameworks (MOFs) have great potential for sensing applications. Interestingly, the surface area (200–10400 m^2^/g) of MOFs is several times higher than other porous materials (Sarker et al., 2018). These structures have been used in various fields, such as chemical and physical sensing, bio-catalysis, heterogeneous catalysis, imaging, drug delivery, gas storage, separation, and purification (Khezerlou et al., 2023, Xu et al., 2019). Some of the outstanding properties of MOFs are high porosity (up to 90% free volume), high flexible structure, high degree of crystallinity, adjustable pore sizes (0–3 nm, up to 9.8 nm), easy-to-made, good water-common solvents stability, desirable thermal stability (to 773 K), satisfactory photo-catalytic activity, facile outer-surface modification and simple coupling with other materials (Helal et al., 2017, Jahangiri-Dehaghani et al., 2020, Zhou et al., 2019). Lately, there have been several reports of FL MOFs developed for the recognition of TC. For example, Li et al. (2019) used the 2-aminoterephthalic acid (H_2_ATPA) as linkers to coordinate with Al^3+^ ions for the detection of TC in milk samples. Li et al. (2021) reported a ratiometric luminescent non-anuclear Tb-L1 probe for the detection and discrimination of TC from doxycycline, oxytetracycline and chlortetracycline.

In line with these aims, the Cr(III)-MOF-based fluorescent on/off system was easily prepared by mixing the 2-amino terephthalic acid solution and chromium nitrate solution by hydrothermal method. This system shows good water stability (60 days), specific detection ability, high sensitivity (LOD: 0.78 ng/mL), selectivity, and short reaction time for TC detection. Finally, this sensing probe was successfully used to the determination of TC in chicken meat and egg samples with good recovery (95.17 – 106.93 %).

## 2 Material and methods

### 2.1 Reagents

Chromium (III) nitrate hydrate (Cr (NO_3_)_3_·9H_2_O), sodium hydroxide pellets, TC, chloramphenicol (CLP), penicillin (PC), tylosin (TY), enrofloxacin (ENR), erythromycin (ERT), vancomycin (VAN), doxycycline (DOX), and diclazuril (DIC)), were purchased from Sigma-Aldrich Co. LLC. (USA). 2-aminoterephthalic acid (H_2_ATPA), acetonitrile, and Tris Buffer were purchased from Merck Co. (Germany). All of the working solutions were prepared with ultrapure water. The egg and chicken meat samples were purchased from the local market of Tabriz, Iran. All reagents and solvents (analytical grade) in this work were commercially available and used as received.

### 2.2 Synthesize of Cr(III)-MOF sensing probe

The FL sensing probe was synthesized via the hydrothermal method referring to the previous studies with some modifications (Zhang et al., 2019). Firstly, at 23 ± 2 °C, 1.6 g Cr(NO_3_)_3_·9H_2_O, 0.72 g H_2_ATPA, and 0.4 g NaOH were dissolved in 30 mL distilled water, by magnetically stirring (30 min), and then the solution was sonicated for 30 min. After that, the mixture was put in a Teflon-lined autoclave and heated at 150 °C for 1 day. The green precipitate was separated through centrifugation (6000 rpm, 5 min), rinsed with ethanol five times, and then dried at 60 °C to acquire Cr(III)-MOF powders.

### 2.3 Instruments

X-ray diffraction (XRD) patterns of powder were obtained by a Bruker binary V3 diffractometer (Germany) in the range of 5–70°. To confirm sensing probe synthesis, infrared spectra of the ligand and probe were scanned in the range of 400 to 4000 cm^−1^ and recorded using the Bruker-Tensor 27 spectrophotometer (Germany). The Quanta 450 (FEI, USA) scanning electron microscope (SEM) and Quanta 250 (USA) energy-dispersive X-ray spectroscope (EDX) were employed for surface morphology and elemental composition of the sensing probe. The Brunauer − Emmett − Teller (BET) surface area and pore size of sensing probe were measured using a Belsorp mini II (Japan) system after degassing at 120 °C for 2 h. A Thermogravimetric analysis (SDT Q600 analyzer, USA) was used to measure thermal stability. The sensing probe was heated ranging from 45 to 600 °C at a scanning rate of 10 °C/min under air. The UV–VIS absorption spectra of the TC solution were performed on a UV–VIS spectrophotometer, Ultrospec 2000 (Pharmacia Biotech, UK) equipped with 1 cm quartz cells. The Jasco® FP-750 spectrofluorimeter with 50 w xenon lamp (Kyoto, Japan), with 1.0 cm quarts’ cell was used to perform all the fluorescence measurements.

### 2.4 Fluorescence response to TC

Before using of FL sensing probe to detect TC, the experimental parameters including sensor concentration (10–50 mg/L), and pH (3–12), incubation time (0–10 min) were optimized for obtaining the best results. In a typical experimental method, sensing probe (30 mg/L) solution was mixed with different concentrations of TC standard solution (1 mg/L) and then the system was diluted with Tris-HCl buffer (pH = 10.0, 0.01 M) to 2 mL. After incubating for 1 min (reaction MOF with TC), the FL spectra were measured at λ_em_ = 410 nm under an λ_ex_ at 300 nm. The selectivity study for TC was similarly performed upon exposure to interferential substances or other antibiotics. Antibiotics (100 µL, 25 ng/mL) including CLP, PC, TY, ENR, ERT, VAN, DOX, and DIC, metal ions (100 µL, 25 ng/mL) including K^+^, Ca^2+^, Na^+^, Mg^2+^, Zn^2+^ (100 µL, 25 ng/mL), and other substances including glucose, lactose, histidine, and ascorbic acid (AA) were added in Cr(III)-MOF solution respectively, in a procedure similar to that of TC.

### 2.5 Analysis of TC in food samples

For the preparation of chicken meat samples, 1 g sample became homogenized and mixed with 10, 25, and 50 ng/mL of TC standard by vortex for 5 min in 10 mL NaOH (0.1 M). The separated solution was centrifuged at 8000 rpm for 10 min, filtered by filter paper, and diluted 10 times in 0.1 M NaOH (Xu et al., 2020). Before extraction, egg samples were stirred for 20 min to blend egg albumen and egg yolk homogeneously. Then 1 g whole and homogenized egg were spiked and mixed with 50, 100, and 150 ng/mL of TC standard in a 50 mL acetonitrile (80% v/v) using a blender at high speed for 5 min. Then, the samples of spiked eggs were collected and transferred into a volumetric flask to reach 250 mL with distilled water. The final solutions were filtered using a 0.22 µm membrane filter (Wei et al., 2016). Briefly, 300 µL of Cr(III)-MOF (30 mg/L) and 50 μL of spiked samples were added to a 2 mL microtube and reached volume by Tris-buffer (pH = 10.0).

## 3 Results and discussion

### 3.1 Structure characteristics of Cr-H_2_ATPA MOF

**Fig. 1:**
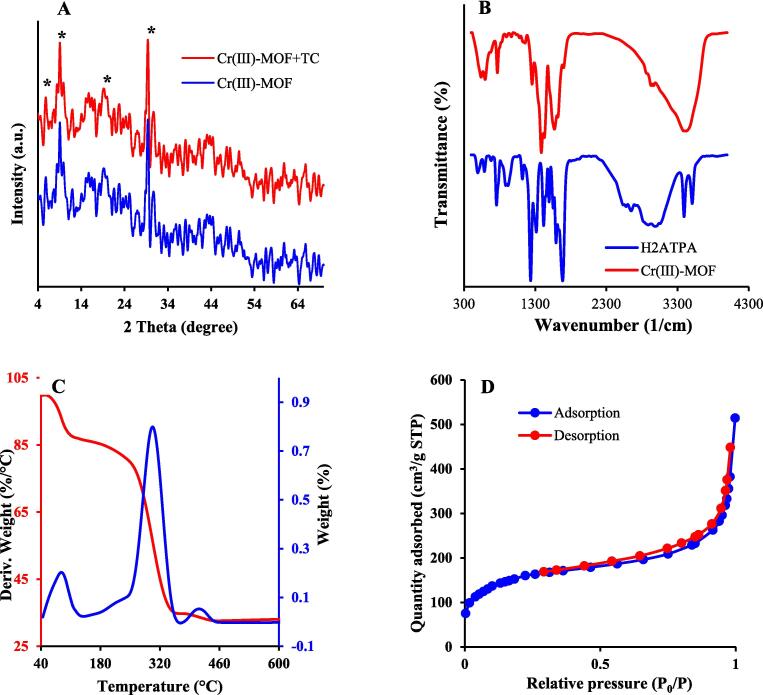
(A) XRD pattern; (B) FTIR spectra, (C) Thermal gravimetric analysis (D) N_2_ adsorption/desorption isotherms of sensing probe at 77 K.

XRD analysis was performed to confirm crystallinity structure and the formation of sensing probe. As illustrated in Fig. 1**A**, the XRD spectrum of fabricated sensing probe coordinated well with other reports (El-Dafrawy et al., 2020, Férey et al., 2005, Rostamnia and Mohsenzad, 2018). The appeared peaks around 2θ = ∼5°, ∼10°, ∼17°, and ∼30°, confirms the successful formation of sensing probe structure. The IR spectra of H_2_ATPA and sensing probe were shown in Fig. 1**B**. The absorption peaks at 3506.08 cm^−1^ and 3391.94 cm^−1^ in H_2_ATPA both vanished and observed a new one at 3419.41 cm^−1^ in sensing probe, corresponding to the —OH and —NH stretching vibration from water absorption (Lin et al., 2012). The wide —OH characteristic peak (2570–2650 cm^−1^) of —COOH in H_2_ATPA is not seen in sensing probe, indicating the complete deprotonation of H_2_ATPA in the structure. The C=O stretching vibration absorption peak at 1698 cm^−1^ in sensing probe shows a sign of weakening and shift of 18 cm^- 1^ compared with that of H_2_ATPA (1680 cm^−1^). The symmetric stretching vibration of C=O at 1236 cm^−1^ in the H_2_ATPA ligand was shifted to 1386 cm^−1^ in sensing probe, indicating the successful coordination of Cr atoms with the carboxylic groups (Iacomi et al., 2022). According to the TGA profiles, sensing probe undergoes a three-stage thermal decomposition during heating (Fig. 1**C**). The first stage occurred at around 88.50 °C, owing to the evaporation of water and any other residual solvents. The second stage occurred around 302.77 °C due to the decomposition of the organic ligand. In the third stage, decomposition of inorganic compounds ocurs at 412.70 °C. After that, the weight of the sensing probe does not change (Xie et al., 2015). Also, the weight loss in the range of 45–600 ℃ for is 66.98%. From Fig. 1**D**, BET surface areas, and pore volume of the sensing probe are assessed at 541.94 m^2^/g and 0.71 cm^3^/g by N_2_ adsorption–desorption isotherms (P/P_0_ = 0.99) at 77 K, respectively. The pore diameter of the probe was 5.26 nm, which indicates the mesoporous structure existed in the sensing probe.

**Fig. 2:**
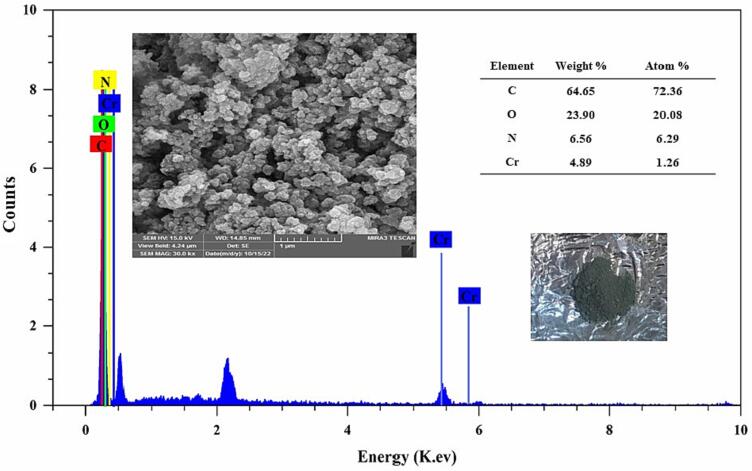
SEM and EDX spectra of sensing probe. The inset shows the photo of Cr-MOF green powder, and table of chemical composition. (For interpretation of the references to colour in this figure legend, the reader is referred to the web version of this article.)

The morphology of the sensing probe was characterized by SEM. As seen in Fig. 2, the sensing probe revealed a reticular structure. The EDX of the sensing probe reveals that C, N, O, and Cr can be detected in this sensor. The chemical composition of the sensing probe is C: 64.65, O: 23.90, N: 6.54, and Cr: 4.89 % (Table inserted in Fig. 2). The FL excitation and emission spectra of sensing probe were recorded in an aqueous solution. When excited at 300 nm, the sensing probe displays a wide FL band in the range of 300–500 nm with the maximum emission at 410 nm.

### 3.2 Optimization of the experimental measurements

**Fig. 3:**
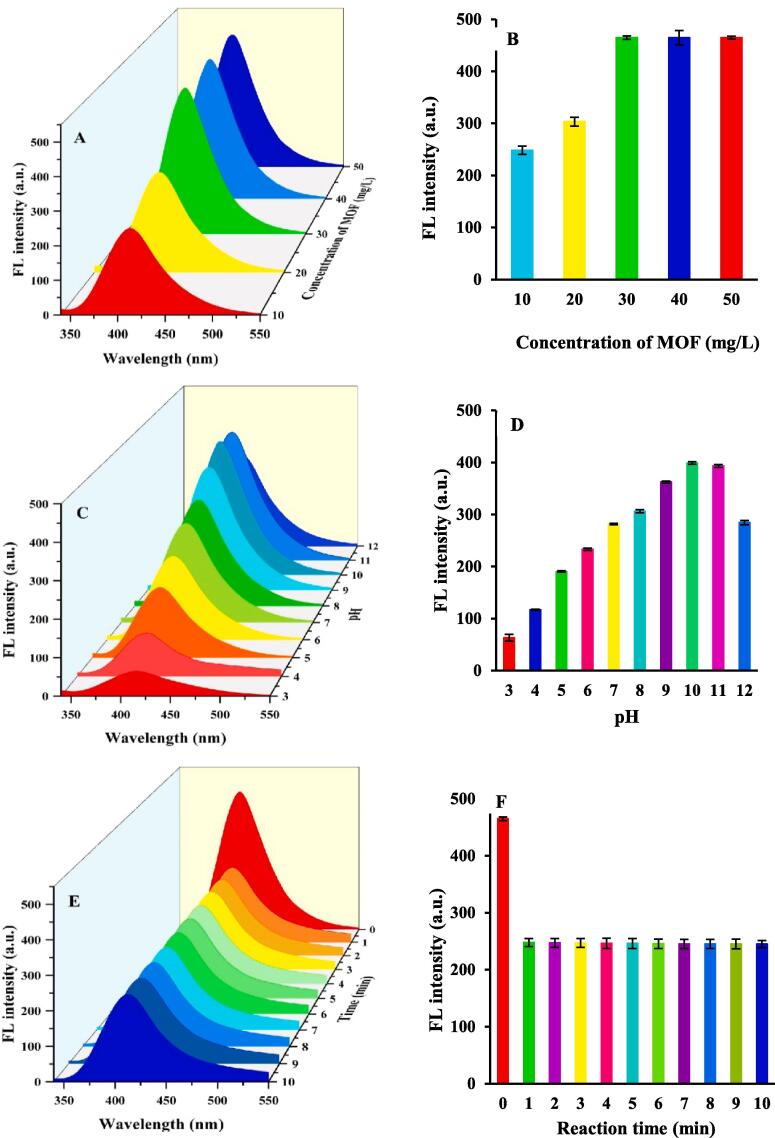
Optimization of the experimental measurements (A and B) sensing probe concentration; (C and D) pH values of the system; (E and F) reaction time.

To evaluate the potential of the sensor, different parameters that could affect the detection efficiency of the probe system, which includes the concentration of the sensing probe, pH of the detection matrix, and reaction time, were then studied in detail and optimized. As shown in Fig. 3**A and B,** the amount of sensing probe was tested from 10 to 50 mg/L. The FL intensity of the sensing probe was increased with increasing the amount of sensing probe from 10 to 30 mg/L. When the amount of the sensing probe was more than 30 mg/L, the FL intensity of the sensing probe was relatively stable. The pH plays the main role in the detection of TC. The pH values were optimized by the Tris-buffer solution range from 3 to 12. Such as Fig. 3 **C and D** show that with the increase of pH value, the FL intensity sensing probe gradually increased until the pH values reached 10.0. When the pH value was more than 10, the FL intensity of sensing probe decreased. Hence, sensing probe showed high FL intensity in solution with pH = 10.0 (Alizadeh Sani et al., 2023, Li et al., 2019). The reaction time between the sensing probe (30 mg/L) and TC (25 ng/L) was evaluated. As shown in Fig. 3 **E and F**, the FL intensity of sensing probe at 410 nm remains almost constant after 1 min, indicating the FL stability of the MOF. When TC was added, the FL intensity declined by 87 % in 1 min, and the FL intensity reached a constant amount after 1 min, revealing that TC is fully combined with the active sites of the sensing probe (Li et al., 2019). These results exhibited that the sensing probe was a “rapid response” FL probe for the detection of TC.

Hence, the amount of the sensing probe, pH, and reaction time were selected to be 30 mg/L, 10.0, and 1 min, respectively; to obtain the best results in the subsequent experiments.

### 3.3 Analytical approach

**Fig. 4:**
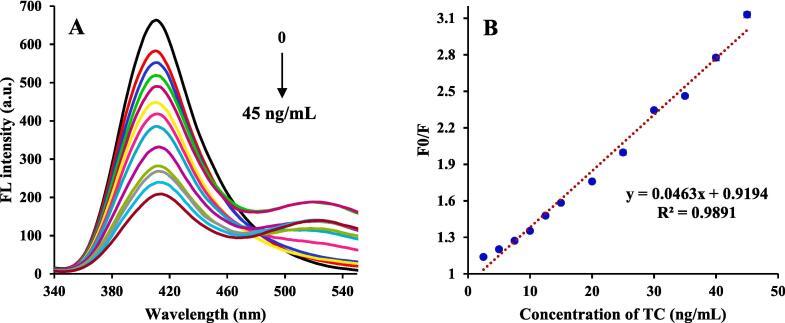
(A) The FL spectra (λ_em_ = 410 nm) of ensing probe showing quenching in the presence of different concentrations of TC, (B) The S–V plot of sensing probe quenched by TC.

In the sensing experiment, the detection performance of the sensing probe toward TC was determined based on optimization conditions. After the addition of different concentrations of TC to the sensing probe solution, the FL intensity of the sensing probe solution at 410 nm decreased gradually as the TC content increased, as shown in Fig. 4**. A and B.** The reduction of FL intensity may be due to Förster Resonance Energy Transfer, and inner-filter effect. The equation of linear regression of the sensing probe towards TC was also described as y = 0.0463x + 0.9194, with an excellent linear relationship at the concentration range of 5–45 ng/mL. The K_sv_ value was 2.11 × 10^-3^ mL/ng. Based on LOD = 3 σ/S (σ is the standard deviation of the blank sample (n = 12), and S is the slope of the calibration curve), the calculated LOD was 0.78 ng/mL. The LOD value is lower than the maximum residue limit of TC in muscle and egg adjusted by the EU (Baghani et al., 2019, Yang et al., 2021). The sensing data of recent studies for TC measuring are tabulated in Table 1, which presents the high sensitivity of the sensing probe toward the TC antibiotic. In a MOF fluorescent assay, Liu et al. (2021) designed Tb-MOF probes for TC sensing using simple one-pot hydrothermal. The Tb-MOF fluorescent assay was found to have an LOD of 81.8 ng/mL, which was higher than the LOD of our study. In addition, a fluorescent aptasensor was developed by L. Zhang et al. (2020) to detect TC based on the fabrication of aptamers-functionalized nitrogen-doped graphene quantum dots coupled with cobalt oxyhydroxide nanoflakes. Their designed aptasensor was able to obtain a LOD of 0.95 ng/mL to identify TC. Also in another study, Hu et al. (2021) fabricated FL sensor for ultrasensitive detection of TC through the encapsulation of Mg and N co-doped carbon dots into the molecularly imprinted polymer. They reported an LOD of 0.79 ng/mL for TC in milk, egg, and pork samples, which was the same as our LOD. However, our method needs much lower reagents, and it is cost-effective and simpler with high sensitivity.

**Table 1:** Comparison of different methods for sensing of TC.

**Detection Method**	**Sensing materials**	**Analytical parameters**	**Food sample**	**Ref.**
Fluorescence	Aptasensor	LR: 1–100 ng/mL	Egg	(L. Zhang et al., 2020)
		LOD: 0.95 ng/mL		
Fluorescence	ZIF-8&*N*-CDs@MIP	LR: 100–4000 ng/mL	Milk, egg	(Zhang et al., 2023)
		LOD: 45 ng/mL		
Fluorescence	Eu-MOF	LR: 0–140 μM	Milk	(Z. Gan et al., 2021)
		LOD: 39.8 nM		
Fluorescence	NH_2_-MIL-53(Al)	LR: 0.00–72.33 μM	Milk	(Li et al., 2019)
		LOD: 26.16 nM		
Fluorescence	Tb-MOF	LR: 0–44.4,	Milk	(Liu et al., 2021)
		44.4–108.4 ng/mL		
		LOD: 81.8 ng/mL		
Luminescent	Nitrogen-doped carbon quantum dots	LR: 0–100 μM	Rat serum and milk	(Wang et al., 2022)
		LOD: 0.344 μM		
Colorimetric	Gold nanoclusters	LR:1–16 μM	Liquid milk and solid milk	(Z. Zhang et al., 2020)
aptasensor		LOD: 46 nM		
Fluorescence	Nitrogen-doped carbon dots	0.2–56.0 μM	Honey, milk, fish and pork	(Jia et al., 2023)
		LOD: 33.8 nM		
Fluorescence	Molybdenum disulfide nanoplates	LR: 0–50 μM	Milk, milk powder and bovine muscle	(Jia et al., 2019)
		LOD: 0.032 μM		
Electrochemical	GCE modified with AuNPs and multi-walled carbon nanotubes	LOD: 42 ng/mL	Chicken meat, egg	(Palisoc et al., 2019)
Fluorescence	Mg/N co-doped CDs -MIP	LR: 5–100 ng/mL	Milk, egg, and pork	(Hu et al., 2021)
		LOD: 0.79 ng/mL		
HPLC	–	LR: 50–5000 ng	Chicken meat and liver	(Shalaby et al., 2011)
		LOD: 5 ng		
UPLC-FLD	–	LR: 35.2–500.0 μg/kg	Chicken muscle	(He et al., 2022)
		LOD: 6.1 μg/kg		
Fluorescence	Cr(III)-MOF	LR: 5–45 ng/mL	Chicken meat, egg	Present work
		LOD: 0.78 ng/mL		

CDs: Carbon dots; GCE: glassy carbon; electrode UPLC-FLD: ultra-performance liquid chromatography-fluorescence detection; LOD: limit of detection; LR: linearity range.

### 3.4 Selectivity, stability, and reproducibility

**Fig. 5:**
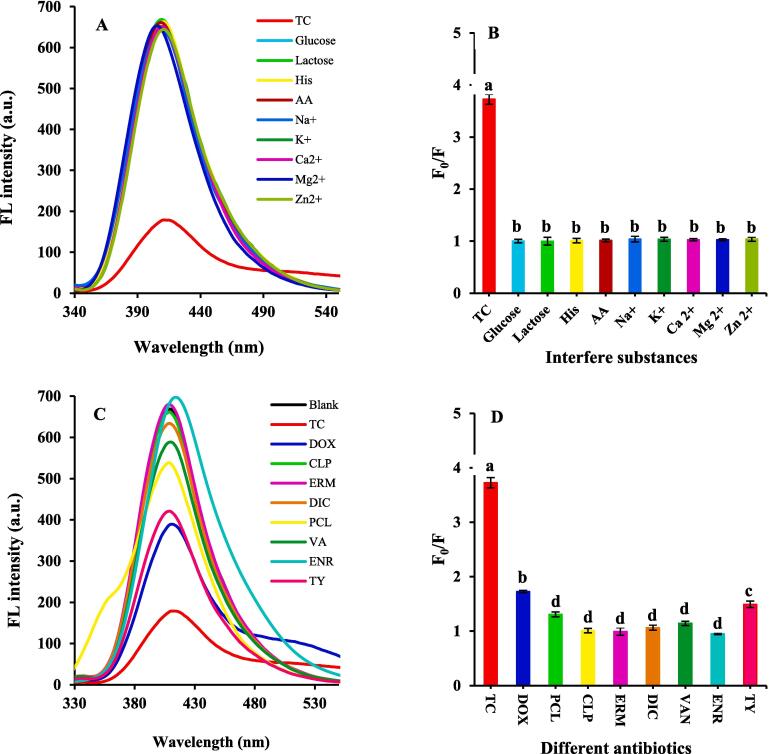
The FL spectra and F_0_/F of the sensing system in the presence of ions and physiological substances (A and B), in the presence of 25 ng/mL antibiotics (C and D).

In addition to sensitivity, the selectivity of a sensing probe is an important parameter for specific analyte detection in real samples. Due to the complexity of the real samples detection, coexisting antibiotics, common ions (Na^+^, K^+^, Mg^2+^, Ca^2+^, Zn^2+^), and physiological substances (His, AA, Glucose, Lactose) might react with the MOF or TC, and interfere the detection of TC. As seen in Fig. 5 **A-D,** only TC (25 ng/mL) is conducive to FL quenching at 410 nm, and the quenching efficiencies (F_0_/F) of sensing probe for the antibiotics follow the order of TC > DOX, which may be related to the similar molecular structure of TC and DOX. Because they are in tetracycline-class antibiotics. Whilst the effects of other substances on sensing probe FL intensity were negligible. These results confirm that the sensing probe has high selectivity toward TC. The detection stability is a very crucial parameter for the practical application of sensing probes. To evaluate the water stability of the sensing probe, we evaluated the FL intensity at different times from 0 to 60 days. The sensor displays alluring FL stability for 60 days without obvious FL intensity loss. Therefore, the sensing probe has shown desirable stability in the water. The reproducibility and repeatability of the Cr(III)-MOF sensing probe have been evaluated intra- and inter-day precision basis. Under optimum conditions, the inter- and intra-day precision has been assessed at the 25 ng/mL concentration of TC. Intera-day stability of the sensing probe has been performed by FL method five replicates during one day at the same interval times. The FL intensity was insignificantly decreased. Intera-day stability of the sensing probe has been performed by FL method five replicates during one day at the same interval times. The FL intensity was almost unchanged (n = 5, RSD = 0.99%) (Fig. S1A). Moreover, inter-day precision of the probe was performed at three replicates on seven different days. As seen from Fig. S1B, the FL intensity was insignificantly decreased (n = 7, RSD = 1.0%). The designed Cr(III)-MOF sensing probe was stable enough.

### 3.5 Measuring of TC in chicken meat and egg samples

To validate the reliability and applicability of the sensing probe for TC detection in chicken meat and egg samples, spike and recovery tests were performed. The results of recovery tests for spiked TC in different samples were summarized in Table S1. The concentration of TC in the chicken meat sample was found to be 9.51, 25.07, and 51.89 ng/mL (n = 3) and the recoveries were 95.17 to 103.78 % with relative standard deviations (RSD) of < 4.6%. The results were in agree with the value sensing probe (10, 25, and 50 ng/mL). The concentration of TC in the egg sample was found to be 50.75, 106.93, and 149.93 ng/mL (n = 3) and the recoveries were 99.96 to 106.93 % with RSD of < 6.4%. The results were in agreement with the value sensing probe (50, 100, and 150 ng/mL). These results indicated that the probe possessed good-pleasing results for TC determination in a food sample.

## 4 Conclusion

A novel amine-functionalized Cr(III)-MOF has been synthesized via a simple one-step hydrothermal method and was used as a “turn-off” sensor for TC detection in chicken meat and egg samples. The sensing probe showed good water stability, a fast response time (1 min), a broad linear range (5 – 45 ng/mL), and high selectivity toward TC. The LOD for TC was 0.78 ng/mL. Moreover, the estimated recoveries of TC in chicken meat and egg ranged from 95.17 to 106.93 % and RSD (n = 3) was <6.4 %. It can be concluded that a sensing probe could be used for ultrahigh sensitive determination of TC in commercial samples due to simplicity, stability, robustness, reliability, and user-friendly. As a result, it appears that the sensor system described in this paper is excellent at detecting TC present at low levels in food. This is the first Cr(III)-based metal–organic framework fluorescence sensor for the detection of tetracycline in diverse meals that has been reported to our knowledge. Furthermore, the findings demonstrated that the system can precisely gauge the impact of various operational parameters on the stability of the system as planned under various conditions (pH and time) to detect TC concentration. The outcomes of the current study can be applied to other food samples and environmental research.

## CRediT authorship contribution statement

**Arezou Khezerlou:** Methodology, Validation, Formal analysis, Investigation, Data curation, Writing – original draft, Writing – review & editing, Visualization. **Milad Tavassoli:** Methodology, Formal analysis, Investigation, Data curation, Visualization. **Mahmood Alizadeh Sani:** Methodology, Validation, Formal analysis, Investigation, Data curation, Writing – review & editing. **Zahra Ghasempour:** Conceptualization, Methodology, Validation, Investigation, Writing – review & editing. **Ali Ehsani:** Resources, Writing – review & editing, Supervision, Funding acquisition. **Balal Khalilzadeh:** Conceptualization, Methodology, Validation, Formal analysis, Investigation, Data curation, Writing – review & editing, Visualization.

## Declaration of Competing Interest

The authors declare that they have no known competing financial interests or personal relationships that could have appeared to influence the work reported in this paper.

## Data Availability

Data will be made available on request.
