# Supplementary material for: Rapid and sensitive detection of tetracycline residue in food samples using Cr(III)-MOF fluorescent sensor
Source: Food Chem X. 2023 Sep 16;20:100883. doi: 10.1016/j.fochx.2023.100883 (PMC10740053; doi:10.1016/j.fochx.2023.100883)
Supplement: Supplementary data 1 [file mmc1.docx]

Supplementary Materials

**Rapid and sensitive detection of tetracycline residue in food samples using Cr(Ⅲ)-MOF fluorescent sensor**

Arezou Khezerlou ^1,2˦^, Milad Tavassoli ^1,2˦^, [Mahmood Alizadeh Sani](https://sciprofiles.com/profile/1595355) ^3^, Zahra Ghasempour ^4^, Ali Ehsani ^4*^, Balal Khalilzadeh ^5**^

*^1^ Student Research Committee, Department of Food Science and Technology, Faculty of Nutrition and Food Science, Tabriz University of Medical Sciences, Tabriz, Iran.*

*^2^ Biotechnology Research Center, Tabriz University of Medical Sciences, Tabriz, Iran.*

*^3^ Division of Food Safety and Hygiene, Department of Environmental Health Engineering, School of Public Health, Tehran University of Medical Sciences, Tehran, Iran.*

*^4^ Department of Food Science and Technology, Faculty of Nutrition and Food Sciences, Tabriz University of Medical Sciences, Tabriz, Iran.*

*^5^ Stem Cell Research Center (SCRC), Tabriz University of Medical Sciences, Tabriz 51666-14711, Iran.*

*˦ equal first author*

**Corresponding authors**

Ali Ehsani, PhD., Department of Food Science and Technology, Faculty of Nutrition and Food Sciences, Tabriz University of Medical Sciences, Tabriz, Iran. Tel: +984133357581-3; Fax: +984133340634, Email: [ehsani@tbzmed.ac.ir](mailto:ehsani@tbzmed.ac.ir)

Balal Khalilzadeh, Ph.D., Stem Cell Research Center (SCRC), Tabriz University of Medical Sciences, 51664-14766 Tabriz- Iran, Tel: +98(41)-33363311; Fax: +98(41)33363231. Email: [balalkhalilzadeh@gmail.com](mailto:balalkhalilzadeh@gmail.com), [khalilzadehb@tbzmed.ac.ir](mailto:khalilzadehb@tbzmed.ac.ir)


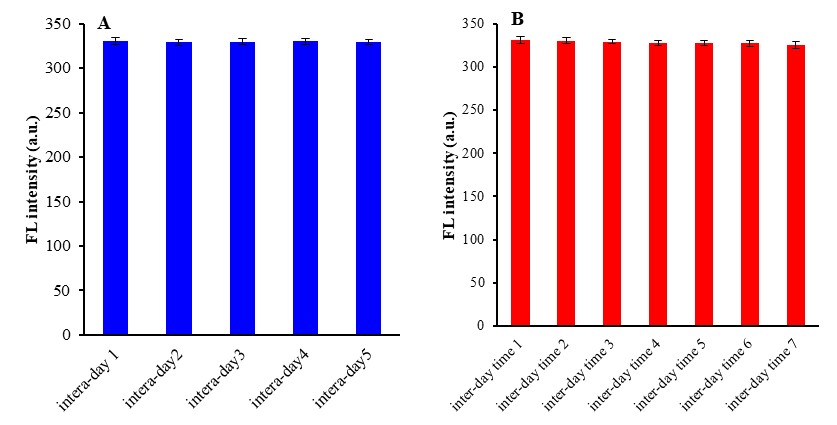


**Fig. S1**. The (A) repeatability and (B) reproducibility experiments. The response of Cr(Ⅲ)-MOF sensing probe to 50 ng/mL TC.

| **Table S1.** Recovery test of TC spiked in chicken meat and egg samples | | | | |
| --- | --- | --- | --- | --- |
| **Samples** | **Spiked TC (ng/mL)** | **Found TC** | **Recovery (%)** | **RSD (%, n=3)** |
| **Chicken meat** | 0 | - | - | - |
|  | 10 | 9.51 ± 0.44 | 95.17 | 4.6 |
|  | 25 | 25.07 ± 0.45 | 100.26 | 1.8 |
|  | 50 | 51.89 ± 0.65 | 103.78 | 1.3 |
| **Egg** | 0 | - | - | - |
|  | 50 | 50.75 ± 1.25 | 101.52 | 2.46 |
|  | 100 | 106.93 ± 6.88 | 106.93 | 6.44 |
|  | 150 | 149.93 ± 9.37 | 99.96 | 6.25 |
